# Contextual Refinement of Regulatory Targets Reveals Effects on Breast Cancer Prognosis of the Regulome

**DOI:** 10.1371/journal.pcbi.1005340

**Published:** 2017-01-19

**Authors:** Erik Andrews, Yue Wang, Tian Xia, Wenqing Cheng, Chao Cheng

**Affiliations:** 1 Department of Genetics, Geisel School of Medicine at Dartmouth, Hanover, New Hampshire, United States of America; 2 Institute for Quantitative Biomedical Sciences, Geisel School of Medicine at Dartmouth, Lebanon, New Hampshire, United States of America; 3 School of Electronic Information and Communications, Huazhong University of Science and Technology, Wuhan, Hubei, China; 4 Norris Cotton Cancer Center, Geisel School of Medicine at Dartmouth, Lebanon, New Hampshire, United States of America; Ottawa University, CANADA

## Abstract

Gene expression regulators, such as transcription factors (TFs) and microRNAs (miRNAs), have varying regulatory targets based on the tissue and physiological state (context) within which they are expressed. While the emergence of regulator-characterizing experiments has inferred the target genes of many regulators across many contexts, methods for transferring regulator target genes across contexts are lacking. Further, regulator target gene lists frequently are not curated or have permissive inclusion criteria, impairing their use. Here, we present a method called iterative Contextual Transcriptional Activity Inference of Regulators (icTAIR) to resolve these issues. icTAIR takes a regulator’s previously-identified target gene list and combines it with gene expression data from a context, quantifying that regulator’s activity for that context. It then calculates the correlation between each listed target gene’s expression and the quantitative score of regulatory activity, removes the uncorrelated genes from the list, and iterates the process until it derives a stable list of refined target genes. To validate and demonstrate icTAIR’s power, we use it to refine the MSigDB c3 database of TF, miRNA and unclassified motif target gene lists for breast cancer. We then use its output for survival analysis with clinicopathological multivariable adjustment in 7 independent breast cancer datasets covering 3,430 patients. We uncover many novel prognostic regulators that were obscured prior to refinement, in particular NFY, and offer a detailed look at the composition and relationships among the breast cancer prognostic regulome. We anticipate icTAIR will be of general use in contextually refining regulator target genes for discoveries across many contexts. The icTAIR algorithm can be downloaded from https://github.com/icTAIR.

## Author Summary

Gene expression regulators, such as transcription factors and microRNAs, are critical actors in cellular physiology and pathophysiology and act by modulating the expression levels of sets of target genes. Given their significance, numerous experiments have sought to characterize the specific target genes of specific regulators, which in turn has led to regulator target gene list databases. Unfortunately, these lists are plagued by poor curation and validation. Further, all lists suffer from the fundamental issue that regulator targets vary across tissue type and physiological state, or “context”, making them poor for conducting downstream, context-specific analyses. To address this issue, here we present a method called icTAIR that contextually-refines regulator target gene lists. To demonstrate its value, we use icTAIR to take the largest-available database of regulator target gene lists, refine it for the breast cancer context, and use both the pre-refined and refined lists for downstream survival analyses in over 3,400 tumors. We find that icTAIR improves the statistical power of the analyses by multiple orders of magnitude. This in turn lets us map the relational network of breast cancer regulators and identify regulators with prognostic effects even after clinicopathological adjustment. We anticipate icTAIR will be broadly useful in regulator studies.

## Introduction

The major gene expression regulators, DNA-binding transcription factors (TFs) and mRNA-binding microRNAs (miRNAs), have long been known to play critical roles in cellular physiology and pathophysiology, especially cancer [1–4]. By modulating transcription (TFs) or complementarily binding to the 3’ UTR of mRNA transcripts and decreasing or silencing their expression (miRNAs), they affect the cellular state by the integration of the induced changes in protein levels of their regulatory targets. Far from monotonous, their targets and functions vary based on the tissue and cellular state (hereafter referred to as the “context”) within which they are expressed. This reflects contextual variability in hetero- and eu-chromatin composition, promoter methylation status, concurrent exogenous regulatory activity, baseline gene expression, and post-translational modifications, among other factors [5, 6].

In a widespread effort to understand their activities, numerous experiments and tools seeking to identify and characterize regulators have emerged over the past decade. Among these, the Encyclopedia of DNA Elements (ENCODE) [7] project has and continues to conduct Chromatin ImmunoPrecipitation followed by Sequencing (ChIP-Seq) studies to explore genomic regions of TF binding, from which TF regulatory target genes can be inferred [8]; many groups have conducted similar experiments leading to databases of TFs and their targets, such as TRANSFAC [9–11], ReMap [12] and ChEA [13]. On the miRNA side, both experimental techniques (knockdowns or inductions, combined with gene expression analysis) and computational methods, like miRanda [14], PicTar [15], PITA [16], RNAhybrid [17], and TargetScan [18], have led to the miRBase [19], TarBase [20–23], and miRTarBase [24, 25] databases of miRNAs and their predicted targets, among others. Still more databases, such as the Molecular Signatures Data Base (MSigDB) [26, 27], compile results across regulators, contexts and other databases.

While these sources of regulators and their targets promise to enhance understanding of regulator activities and their implications, currently the full extent of this promise is unfulfilled. This is due to two intertwined issues: (1) target gene inaccuracy and (2) problems of contextual transference. Without doubt, the accurate determination of regulator target genes is difficult, with much experimental noise and room for identification algorithm improvement [28], leading to high false positive and negative rates of a regulator’s identified target genes. Second, target gene lists are generated and confirmed using context-specific experiments, if experimentally generated and confirmed at all, creating the challenge of transferring and combining results from one context to another. While this challenge has been approached with success through set operation intersection of target gene lists to create “core” target gene sets for use in a new context [29], this approach necessarily drops context-specific target genes, even if genuine, and results in the rapid shrinkage of target gene lists as more and varying contexts are intersected. As such, set operation unions of target gene lists may be performed instead; however, this results in the opposite issue of creating non-specific gene lists that increase in size and inaccuracy as more and varying contexts are combined. All told, these issues highlight the need for a new approach for target gene list refinement and contextual transference.

In this paper, we present a method called iterative Contextual Transcriptional Activity Inference of Regulators (icTAIR) to jointly solve these issues. icTAIR’s key insight is simple: a strong, genuine target of a regulator for a context will have its expression correlate with the regulator’s activity in that context. Put another way, each target gene’s degree of expression should contribute to the degree of a regulator’s activity. icTAIR applies this insight by calculating a regulator’s activity level for a context and correlating it with each of its target genes’ expression levels, thereby discerning strong, genuine targets from false targets for that context. icTAIR then iteratively drops false and/or weak targets from the target gene list and repeats the process until it achieves a stable target gene list. As a crucial additional outcome, this refinement greatly improves the precision of the calculation of a regulator’s activity to enhance downstream analysis.

To validate icTAIR and demonstrate the power of this precision improvement, we employ it to contextually refine the MSigDB c3 database of 825 TF, miRNA, and unclassified regulatory motif target gene lists for breast cancer using The Cancer Genome Atlas (TCGA)’s gene expression dataset of 590 breast cancer (BRCA) samples. First, we use the output of each iteration of icTAIR to see how each regulator’s list of target genes changes across consecutive iterations. Concurrently, we employ these consecutive lists to calculate regulator activity levels and survival analyses in the independently-generated METABRIC dataset [30] of 1992 breast cancer samples, finding that icTAIR refinement creates stable gene lists that markedly improve the analyses. We then use icTAIR’s final output for survival analysis for each of the 825 regulatory motifs in 7 independent datasets covering 3,430 patient samples. We find icTAIR greatly improves our analysis’ resolution and reveals numerous prognostic regulators that were obscured prior to icTAIR refinement. Gains in resolution are especially large for miRNAs. Further validation checks of specific results are passed and give us full confidence in the icTAIR method and results. Next, we follow-up by using these results to look in detail at the composition and relationships among the breast cancer prognostic regulome, and conclude by identifying 29 regulatory motifs that are prognostic in all datasets even after clinicopathological multivariate adjustment. Excitingly, we find that regulatory motifs associated with the E2F and NFY TF families have the greatest and most significant effects on breast cancer prognosis, in alignment with past results for E2F [29] and novel for NFY. Given the strength of these findings, especially in contrast to those achieved without icTAIR refinement, we anticipate icTAIR will be of general use for contextual refinement of regulator target genes and discovery of novel activities and implications across many biological contexts.

## Results

### Overview of icTAIR algorithm

**Fig 1:**
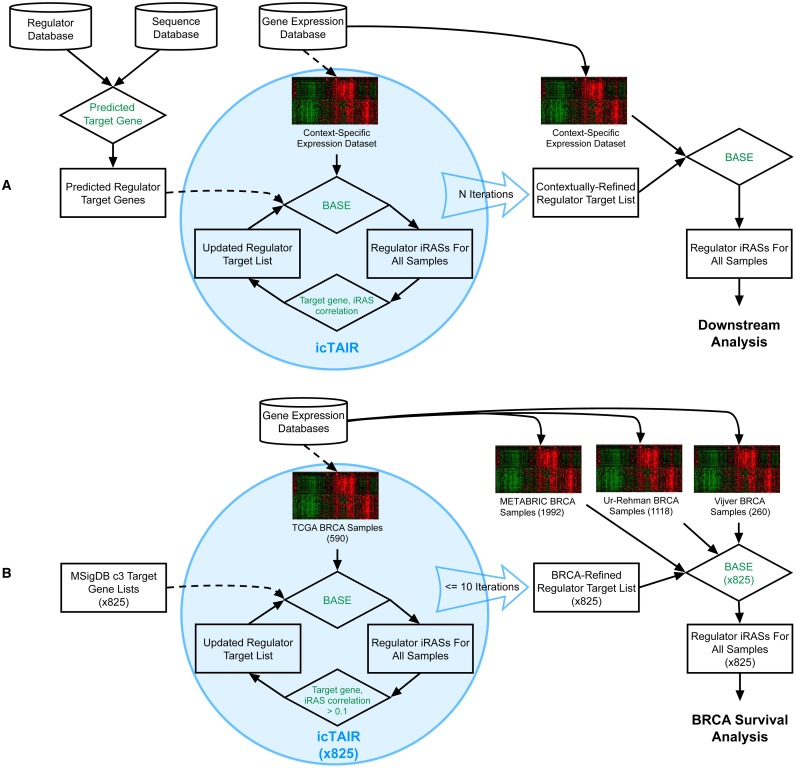
An overview of icTAIR and its use. (A) The general icTAIR workflow. icTAIR is an iterative algorithm (blue oval) that requires as input a regulator’s target gene list and a gene expression dataset for samples in a context of interest. Using the previously defined method BASE ([31]; see Materials and Methods), it integrates the target gene list and gene expression data to infer the activity level of the regulator for the context, quantifying it into an individual Regulatory Activity Score (iRAS) for each sample. For each gene in the regulator’s target gene list, it then calculates the spearman correlation between the gene’s expression level and the iRAS across all samples, creating a new list of correlated target genes (blue oval, left). With this updated list, it repeats the entire process for N iterations until a stable, contextually refined target list is rendered. With this final list, BASE can be once again used with expression data of samples for the context of interest to generate iRASs and enable downstream analysis of the implications of regulator activity. (B): icTAIR as applied here. See Materials and Methods. For both: Rectangles = datasets; rhombi = operations.

A schematic overview of icTAIR and its integration into regulator functional analysis is presented in Fig 1. icTAIR is an iterative algorithm that takes as its input previously-defined target genes of regulators and gene expression data from a dataset of samples for a given context (Fig 1A). It then employs the previously described Binding Association with Sorted Expression (BASE) method [31] to integrate these inputs and calculate individual Regulatory Activity Scores (iRASs) for each sample for that regulator. It then correlates each target gene’s expression with the regulator’s iRAS across the samples, drops the weakly and uncorrelated target genes to create an updated target gene list, and repeats the whole process. Once the number of pre-specified iterations are complete, the resultant contextually-refined target gene lists can be used for final regulator iRAS discernment for any dataset of samples within that context.

The specific use of icTAIR for this work is represented in Fig 1B. Each MSigDB c3 target gene list was contextually refined by icTAIR for the TCGA BRCA two-channel gene expression dataset. icTAIR parameters were default set as follows: minimum spearman correlation, 0.1; minimum target gene list length, 20; maximum number of icTAIR iterations, 10. These parameters were empirically chosen to establish relaxed refinement criteria yet maintain sufficient numbers of target genes for downstream analysis. Once the 10 iterations were completed, the final, BRCA-refined regulator target gene lists were combined using BASE with gene expression data from the three independent datasets of METABRIC (1,992 tumors) [30], Ur-Rehman (1,118 tumors) [32] and Vijver (260 samples) [33] to calculate regulator iRASs for each of the tumors in the datasets. These scores were used for the rest of the analysis.

### Consecutive icTAIR outputs result in stable regulator target gene lists that enhance downstream survival analyses

**Fig 2:**
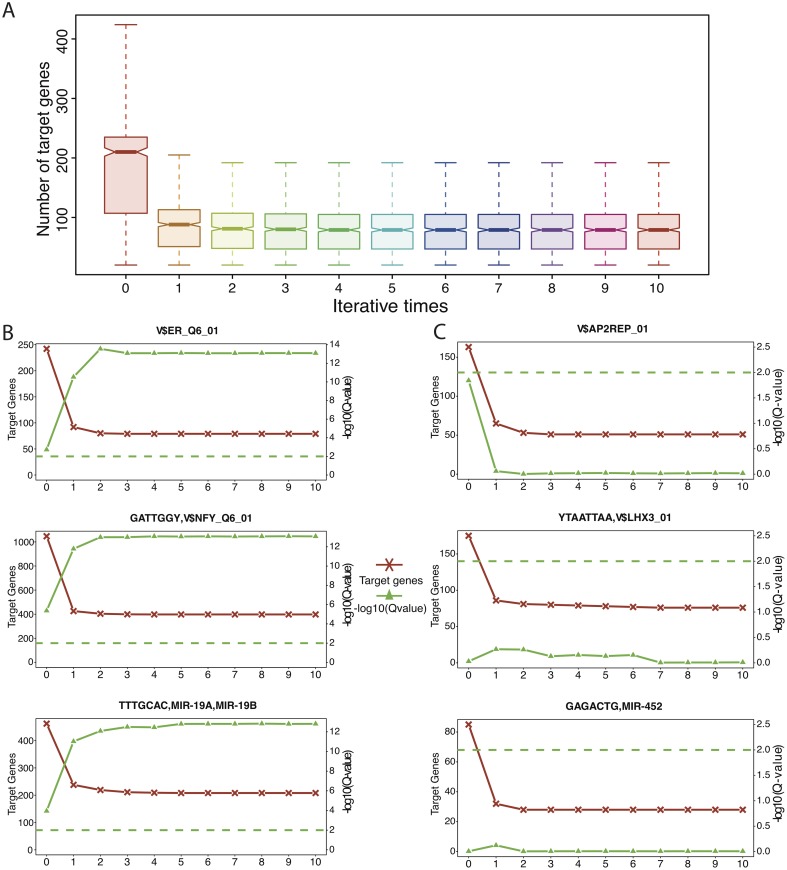
icTAIR consecutive refinement of regulator target gene lists and its effects on downstream analyses. (A) Boxplot of the number of target genes for the regulators in each iteration. Within 2 iterations the plots become relatively homogenous, with stabilization of the regulator target gene lists seen after 5 iterations. (B) Specific target gene list changes and survival analysis results for three most-prognostic regulators, V$ER_Q6_01 (TF), GATTGGY,V$NFY_Q6_01 (Motif), and TTTGCAC,MIR-19A,MIR-19B (miRNA), across the icTAIR iterations. The left y-axis is the number of target genes and the right y-axis is the Q-value of the regulator’s HR, scaled by a–log10 transformation. (C) The same as (B) but for three least-prognostic regulators, V$AP2REP (TF), YTAATTAA,V$LHX3_01 (Motif), and GAGACTG,MIR-452 (miRNA). In contrast to the results of 2B, the Q-values fall in significance with each icTAIR iteration.

To understand the process of icTAIR refinement we investigate the output of each consecutive iteration on the composition of the regulator target gene lists and associated survival analyses. For each icTAIR iteration, we counted the number of target genes on each regulator’s list and plotted how this number evolved across iterations. Fig 2A shows a boxplot of the results for each iteration across the regulators, revealing that the numbers of regulator target genes fall asymptotically and stabilize by the fifth icTAIR iteration. This trajectory matches what one would expect if icTAIR were accurately refining the lists to true contextual targets. To further validate these results, we input each of these regulator target gene lists into BASE using the METABRIC dataset, and then employ the derived iRASs for survival analysis for each regulator for each iteration. To do this, univariate Cox proportional hazards (PH) models were constructed of the consecutive regulator iRASs vs. survival for the samples, yielding Hazard ratios (HRs) and associated p-values that were subsequently corrected for multiple comparisons into Q-values. Ranking these Q-values, we selected three most-prognostic and three least-prognostic regulators (one TF, one miRNA, and one unclassified motif for each group) and plotted how icTAIR refinement iteratively affects each regulator’s number of target genes and Q-value of prognostic significance (Fig 2B and 2C). For both the most (2B) and least (2C) significant regulators, the target gene lists stabilize around 5 iterations, matching the overall results (Fig 2A). Moreover, for the most significant regulators, the Q-values of the results increase in significance with each iteration (Fig 2B), whereas for the least significant regulators, the Q-values decrease in significance (Fig 2C). These results suggest that icTAIR effectively improves the statistical “signal-to-noise” ratios of downstream analyses, as the regulators with initially-significant prognoses see gains in significance and those initially lacking in significance become increasingly insignificant.

### icTAIR contextual refinement uncovers regulator prognostic implications in breast cancer

**Fig 3:**
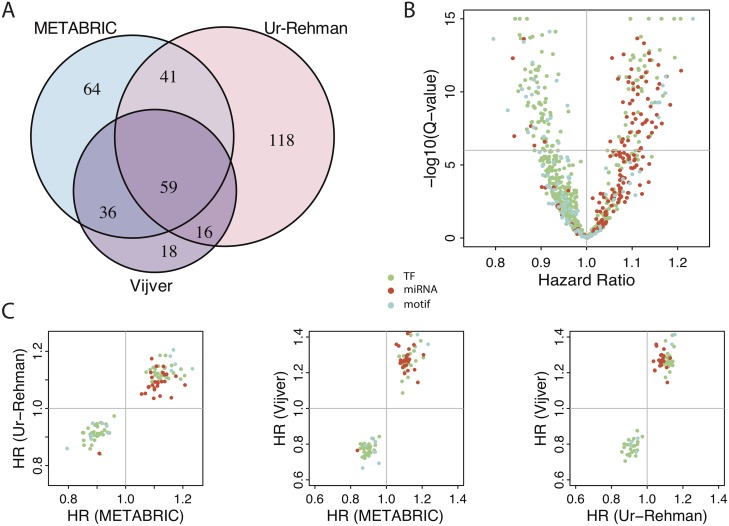
icTAIR used for MSigDB c3 contextual refinement with its output applied to breast cancer survival analysis. (A) Venn diagrammatic breakdown of significant regulators across the datasets. All regulators that passed Q-value significance thresholds (1e-06 for METABRIC and 1e-03 for Vijver and Ur-Rehman) have been tabulated, with the numbers indicating the total amount of regulators for each dataset indicated. 200 significant regulators in METABRIC, 234 in Ur-Rehman, and 129 in Vijver were found, with 59 significant across all three datasets. (B) Volcano plot of the regulators’ Cox PH results in METABRIC Dataset. The x-axis indicates the Hazard Ratio (HR) and the y-axis the FDR-corrected degree of significance (Q-value), scaled by a–log10 transformation. Dots that are higher are more statistically significant, while those that are more polarized to the left or right have a larger survival effect size. The horizontal line indicates a Q-value cutoff of 1e-06. Q-values less than 1e-15 have been censored to that value. (C) Pair-wise analysis of survival effect size concordance between datasets. Left: Ur-Rehmann vs. METABRIC; middle: Vijver vs. METABRIC; right: Vijver vs. Ur-Rehman. Each dot represents a HR coordinate (HR in first dataset, HR in second dataset) for each statistically significant regulator shared between the indicated datasets (*e*.*g*., the left panel includes 100 (41 + 59) regulators (Fig 3A)). Perfect directional (> or < 1) concordance of HRs between all datasets is seen. Dot coloring is by type: green, TF; red, miRNA; cyan, unclassified regulator motif.

To utilize the icTAIR refinement, the final, BRCA-refined target gene lists were inputted into BASE for iRAS calculation into the METABRIC [30], Ur-Rehman [32], and Vijver [33] breast cancer datasets. For each of the regulators, univariate survival analyses were conducted in each dataset. A summary of the results and comparison across datasets is shown in Fig 3. Considering the different sample sizes in the different datasets, we set conservative Q-value thresholds of 1e-06 for the METABRIC (1,992 tumors), 1e-03 for the Vijver (260 samples), and 1e-03 for the Ur-Rehman (1,118 tumors) results to reflect the corresponding differences in statistical power. Using these thresholds, 352 unique survival-associated regulatory programs were found (Fig 3A), of which 59 were significant in all three datasets and deemed pan-dataset univariate prognostic (S1 Table). Examining the makeup of the prognostic regulators, Fig 3B shows the Cox PH results for the 200 regulators in the METABRIC dataset, with each dot a unique regulator color-coded by type and plotted based on its HR and Q-value. Given the dot distribution, it is seen that the TFs (green dots) tend to have more favorable prognostic implications than do miRNAs (red dots). Repeat analyses for the Vijver and Ur-Rehman datasets show similar results (S1 Fig). Further analysis revealed complete concordance of regulator motif HR direction (positive vs. negative) between all pairwise comparisons of the datasets (Fig 3C), indicating that the identified survival-associated regulators have stable pan-dataset prognostic implications (favorable or unfavorable).

To benchmark these results, we repeated this analysis instead using the raw MSigDB c3 target gene lists, with no icTAIR refinement (S2 Fig). Each panel in S2 Fig was drawn with the same parameters (Q-value thresholds and plot areas) as in Fig 3. As seen with the volcano plots (Figs 3B and S2B), Q-value significance of all results is dramatically improved with the use of icTAIR (Fig 3B, smallest Q-value <1e-15; S2B Fig, smallest Q-value >1e-09). Further, without icTAIR the analysis fails to identify any prognostic regulators in the Vijver dataset, resulting in 0 pan-dataset prognostic regulators discovered. Of the datasets with results (METABRIC and Ur-Rehman), the fraction of prognostic regulators that overlaps increases after icTAIR refinement (50% vs. 48% overlap METABRIC, 43% vs. 21% Ur-Rehman, Figs 3A vs. S2A), indicating the improved strength of results. Notably, an examination of the volcano plots shows an especially remarkable improvement in prognosis discernment of miRNAs following the use of icTAIR (Figs 3B vs. S2B, number of red dots above horizontal Q-value threshold line).

### icTAIR-refined results are validated by Estrogen Receptor TF analysis

icTAIR refinement’s biological sensibility was examined via a detailed analysis of the Estrogen Receptor (ER) TF regulatory motif program. MSigDB c3 contains a target gene list for ER named V$ER_Q6_01, for which it was reasoned that a statistically significant, favorable prognosis should be observed in ER+ tumors and no prognostic implications observed for ER- tumors, given that standard breast cancer hormonal therapy targets the ER pathway to great effect and the pathway would be inactive in ER- tumors. If icTAIR refinement is effective and operates as intended, the icTAIR refined V$ER_Q6_01 regulatory program should demonstrate this pattern.

**Fig 4:**
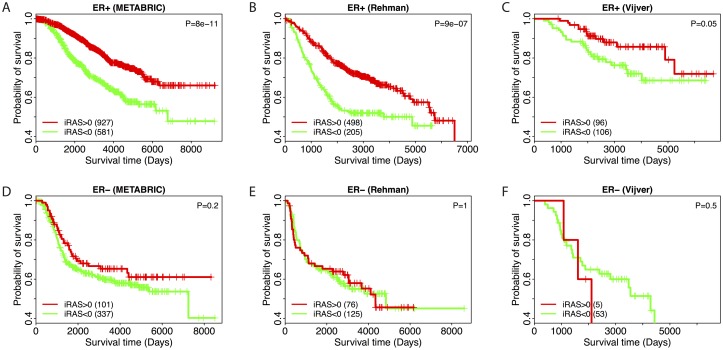
Validation of icTAIR refinement using V$ER_Q6_01. V$ER_Q6_01 iRASs (reflecting ER regulatory activity) were dichotomized around 0 and used to construct Kaplan-Meier survival curves for patient samples stratified by ER staining status (positive versus negative) across all datasets. V$ER_Q6_01 regulatory motif activity confers a favorable prognosis in ER-positive samples across all datasets (A, B, and C, all p-values < 0.05, log-rank test) and has no significant prognostic effect in ER-negative samples (D, E, and F, all p-values > 0.05, log-rank test). Sample numbers for each curve are given in the bottom left and log-rank p-value significance test results in the top right of each panel. Vertical hashes indicate right-censored data points. Survival times are disease-specific for METABRIC, relapse-free for Ur-Rehman, and overall for Vijver, respectively.

The results of this analysis are shown in Fig 4. For construction of all plots, the V$ER_Q1_06 iRASs were dichotomized into two groups around 0, stratified into ER+ and ER- groups based on pathological data annotation, and combined with clinical survival data to construct Kaplain-Meier (KM) curves for each dataset as shown. For both the METABRIC (Fig 4A and 4D) and Ur-Rehman (Fig 4B and 4E) datasets, the ER regulatory motif is highly favorably prognostic within ER+ samples (p-value < 1e-06, log-rank test) but not within ER- ones (p-value = 0.24, METABRIC dataset; p-value = 0.99, Ur-Rehman dataset; log-rank test). For the Vijver dataset (Fig 4C and 4F), the pattern holds but is less pronounced (ER+ p-value = 0.048, ER- p-value = 0.54, log-rank test). Further, the fraction of iRASs>0 is much higher in ER+ vs. ER- samples across all datasets: 2.7, 1.8, and 5.6 times higher for METABRIC, Ur-Rehman, and Vijver, respectively (bottom-left of all panels), consistent with the expectation that ER+ tumors would have greater ER regulatory activity than ER- ones.

### icTAIR-identified pan-dataset univariate prognostic regulators include E2F, HIF1, and MIR-19 motifs

**Fig 5:**
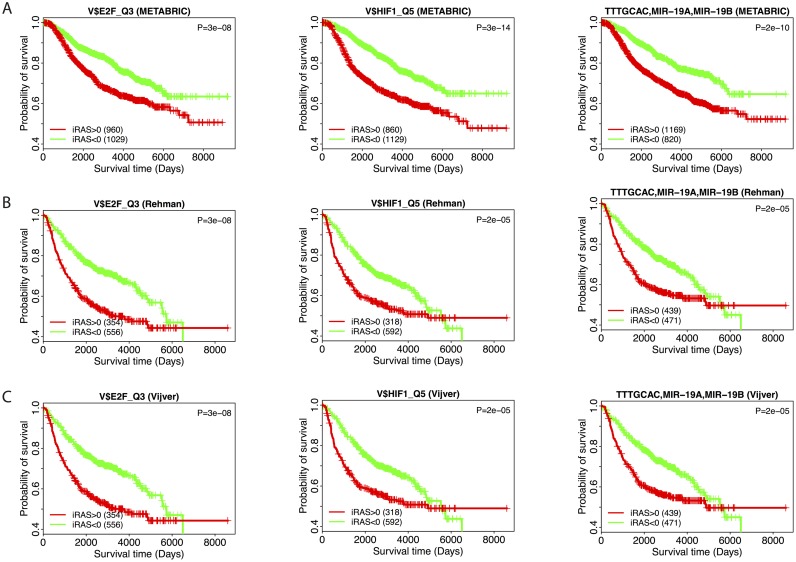
E2F, HIF1, and MIR-19 as examples of prognostic regulators. Among the 59 icTAIR-refined regulatory programs identified as prognostic are E2F_Q3, HIF1_Q5 and the TTTGCAC target sequence of MIR-19 (A and B). Each Kaplan-Meier curve was constructed by dichotomizing the iRASs around 0 for each indicated survival dataset (METABRIC (Fig 5A), Ur-Rehman (Fig 5B), Vijver (Fig 5C)). Increased regulatory activities of these three regulators are found to be associated with worse survival outcomes, in agreement with prior results [29, 34, 35]. Sample numbers for each curve are given in the bottom left and log-rank p-value significance test results in the top right of each panel. Vertical hashes indicate right-censored data points. Survival times are disease-specific for METABRIC, relapse-free for Ur-Rehman, and overall for Vijver, respectively.

Examination of the 59 pan-dataset univariate prognostic regulators (Fig 3B) reveals the presence of both novel and previously identified regulators (S1 Table). Closer analysis of the E2F (a family of TFs), HIF1 (a TF), and MIR-19 (a miRNA) regulatory motifs is provided in Fig 5 as representative members of the group. Dichotomizing each of these regulatory motif’s iRASs around 0 and constructing KM curves for each survival dataset demonstrates the prognostic effects of these three regulators (p-value <0.0001, all regulators and datasets, log-rank test). These results match those from prior studies [29, 34–36].

### Network analysis characterizes the relationships of the icTAIR-identified pan-dataset univariate prognostic regulators

Network analysis was pursued to investigate the relationships among the pan-dataset univariate prognostic regulators. For each regulator, the associated icTAIR-refined target gene lists were examined for the presence of other members in the MSigDB c3 database and used to establish regulator-target relationships. Regulator-redundant motifs were collapsed by regulator and mapped to visualize the regulatory network of the MSigDB c3 regulome.

**Fig 6:**
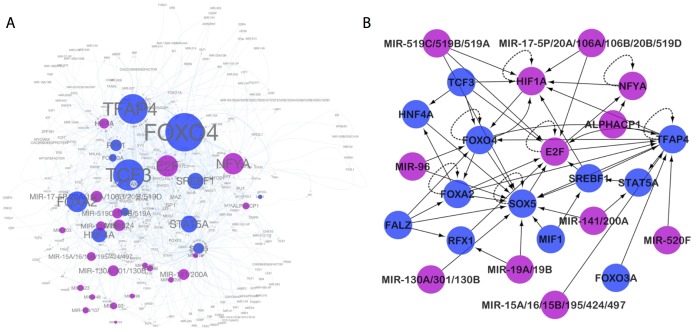
Network analysis of prognostic regulators. For all 59 significant prognostic regulatory motifs, each associated icTAIR-refined target gene list was searched for the presence of other regulators and used to construct a relational map (regulator-to-target) of the prognostic MSigDB c3 regulome. (A) Network map of prognostic regulators, all regulators significant, all regulatees (significant and non-significant) allowed. Size of circle indicates relative number of target regulatees. (B) Network map of prognostic regulators, all regulators and regulatees significantly prognostic. Of note, the unknown regulatory motifs are necessarily excluded here. For both maps: blue dots, HR < 1 of associated regulatory motifs; purple dots, HR > 1 of associated regulatory motifs. Each dot is a composite of regulatory motifs for the same regulator. Arrow directionality -> signifies a regulator -> regulatee relationship. Auto-regulatory relationships are indicated with dotted lines.

Maps of the regulatory relationships of the pan-dataset univariate prognostic regulators targeting all other members of the MSigDB c3 regulome (Fig 6A) and restricted to regulatory relationships among themselves (Fig 6B) are shown. Of note, all unclassified regulatory motifs are necessarily excluded from the right. Size of circles indicate the number of relations of the regulator and color indicates direction of prognosis (blue, more favorable; purple, less favorable).

### 29 regulatory motifs remain prognostic in all datasets after clinicopathological multivariate adjustment

A follow-up multivariate analysis of each univariate prognostic regulator to adjust for clinicopathological factors was pursued. For each of the 59 pan-dataset univariate prognostic regulatory motifs, a multivariate Cox PH model was constructed for it and all available non-redundant clinicopathological factors (see Materials and Methods). Results were corrected for multiple comparisons and all motifs whose hazard ratios were significant at Q-value thresholds (see Materials and Methods) in all datasets were kept to create Table 1, ranked in descending order of HR. For the complete multivariate-adjusted results of the 59 pan-dataset univariate prognosic motifs, see S2–S4 Tables. As each of the listed regulators remains prognostic after full clinicopathological variable adjustment, they all contribute prognostic information beyond clinicopathological variables currently collected as part of breast cancer patient diagnosis and care. Certain regulatory programs appear particularly prognostic: the regulatory motifs associated with the TF families E2F and NFY appear multiple times and are ranked at the top of the list. Comparing these results with Fig 6 puts these prognostic effects into a relational context.

**Table 1:** Pan-dataset univariate prognostic regulatory motifs that survive pan-dataset clinicopathological multivariate-adjusted significance testing.

Regulator	METABRIC			Rehman			Vijver		
	HR (95%CI)	P-value	Q-value	HR (95%CI)	P-value	Q-value	HR (95%CI)	P-value	Q-value
V.NFY_C	1.15 (1.09–1.21)	5.0E-07	2.0E-05	1.1 (1.05–1.15)	1.0E-04	1.0E-03	1.2 (1.07–1.35)	2.0E-03	2.0E-02
GATTGGY_V.NFY_Q6_01	1.06 (1.04–1.09)	4.0E-06	9.0E-05	1.07 (1.04–1.12)	1.0E-04	1.0E-03	1.19 (1.06–1.35)	5.0E-03	2.0E-02
V.E2F_03	1.09 (1.04–1.13)	3.0E-05	5.0E-04	1.08 (1.02–1.15)	9.0E-03	2.0E-02	1.16 (1.04–1.29)	5.0E-03	2.0E-02
V.NFY_01	1.08 (1.04–1.12)	5.0E-05	6.0E-04	1.12 (1.06–1.19)	1.0E-04	1.0E-03	1.25 (1.07–1.46)	6.0E-03	2.0E-02
ACATTCC.MIR.1.MIR.206	1.11 (1.05–1.18)	2.0E-04	1.0E-03	1.07 (1.01–1.14)	3.0E-02	4.0E-02	1.09 (1.01–1.17)	3.0E-02	4.0E-02
TGCTGCT.MIR.15A.MIR.16.MIR.15B.MIR.195.MIR.424.MIR.497	1.05 (1.02–1.08)	1.0E-04	1.0E-03	1.05 (1.01–1.09)	1.0E-02	2.0E-02	1.17 (1.05–1.3)	3.0E-03	2.0E-02
V.E2F1_Q3_01	1.12 (1.06–1.19)	2.0E-04	1.0E-03	1.08 (1.01–1.15)	2.0E-02	3.0E-02	1.32 (1.07–1.62)	9.0E-03	2.0E-02
V.NFY_Q6_01	1.09 (1.04–1.15)	2.0E-04	1.0E-03	1.12 (1.06–1.19)	8.0E-05	1.0E-03	1.21 (1.04–1.39)	1.0E-02	3.0E-02
TTGCACT.MIR.130A.MIR.301.MIR.130B	1.06 (1.03–1.1)	5.0E-04	2.0E-03	1.06 (1.02–1.1)	2.0E-03	7.0E-03	1.13 (1.03–1.25)	1.0E-02	3.0E-02
CATTTCA.MIR.203	1.05 (1.02–1.09)	1.0E-03	3.0E-03	1.07 (1.02–1.13)	6.0E-03	1.0E-02	1.13 (1.02–1.25)	2.0E-02	3.0E-02
TTGTTT_V.FOXO4_01	0.97 (0.96–0.99)	7.0E-04	3.0E-03	0.98 (0.97–0.99)	5.0E-03	1.0E-02	0.91 (0.83–0.99)	3.0E-02	4.0E-02
V.E2F_Q3_01	1.06 (1.02–1.09)	7.0E-04	3.0E-03	1.11 (1.04–1.18)	1.0E-03	4.0E-03	1.16 (1.04–1.29)	7.0E-03	2.0E-02
V.E2F1_Q4_01	1.05 (1.02–1.09)	1.0E-03	3.0E-03	1.11 (1.05–1.19)	8.0E-04	4.0E-03	1.16 (1.04–1.29)	7.0E-03	2.0E-02
V.E2F_Q6	1.06 (1.02–1.1)	1.0E-03	4.0E-03	1.09 (1.03–1.15)	2.0E-03	7.0E-03	1.14 (1.02–1.28)	3.0E-02	4.0E-02
V.STAT5A_04	0.93 (0.89–0.97)	1.0E-03	4.0E-03	0.92 (0.87–0.97)	2.0E-03	7.0E-03	0.81 (0.7–0.95)	8.0E-03	2.0E-02
TTTGCAC.MIR.19A.MIR.19B	1.05 (1.02–1.08)	3.0E-03	5.0E-03	1.06 (1.03–1.1)	9.0E-04	4.0E-03	1.16 (1.05–1.29)	4.0E-03	2.0E-02
V.E2F_Q3	1.05 (1.02–1.09)	2.0E-03	5.0E-03	1.11 (1.05–1.17)	1.0E-04	1.0E-03	1.15 (1.04–1.27)	7.0E-03	2.0E-02
V.E2F_Q4_01	1.04 (1.01–1.06)	2.0E-03	5.0E-03	1.1 (1.04–1.17)	1.0E-03	5.0E-03	1.19 (1.06–1.32)	3.0E-03	2.0E-02
V.E2F_Q6_01	1.05 (1.02–1.09)	2.0E-03	5.0E-03	1.09 (1.02–1.16)	1.0E-02	2.0E-02	1.13 (1.04–1.23)	4.0E-03	2.0E-02
KTGGYRSGAA_UNKNOWN	1.09 (1.03–1.15)	3.0E-03	6.0E-03	1.1 (1.02–1.18)	1.0E-02	2.0E-02	1.27 (1.07–1.5)	6.0E-03	2.0E-02
V.E2F4DP1_01	1.07 (1.02–1.12)	3.0E-03	6.0E-03	1.08 (1.02–1.15)	1.0E-02	2.0E-02	1.17 (1.03–1.32)	1.0E-02	3.0E-02
GACAATC.MIR.219	1.06 (1.02–1.1)	4.0E-03	7.0E-03	1.07 (1.02–1.13)	9.0E-03	2.0E-02	1.17 (1.01–1.35)	3.0E-02	4.0E-02
V.E2F_02	1.06 (1.02–1.1)	4.0E-03	7.0E-03	1.07 (1–1.13)	3.0E-02	4.0E-02	1.15 (1.02–1.3)	3.0E-02	4.0E-02
V.E2F1DP1RB_01	1.06 (1.02–1.11)	4.0E-03	7.0E-03	1.09 (1.04–1.15)	7.0E-04	4.0E-03	1.14 (1.02–1.27)	2.0E-02	4.0E-02
V.E2F1DP1_01	1.06 (1.02–1.1)	5.0E-03	8.0E-03	1.07 (1–1.13)	3.0E-02	4.0E-02	1.15 (1.02–1.3)	3.0E-02	4.0E-02
V.E2F1DP2_01	1.06 (1.02–1.1)	5.0E-03	8.0E-03	1.07 (1–1.13)	3.0E-02	4.0E-02	1.15 (1.02–1.3)	3.0E-02	4.0E-02
V.E2F4DP2_01	1.06 (1.02–1.1)	5.0E-03	8.0E-03	1.07 (1–1.13)	3.0E-02	4.0E-02	1.15 (1.02–1.3)	3.0E-02	4.0E-02
AGCYRWTTC_UNKNOWN	0.94 (0.9–0.98)	6.0E-03	9.0E-03	0.94 (0.89–1)	4.0E-02	5.0E-02	0.86 (0.78–0.96)	6.0E-03	2.0E-02
V.FAC1_01	0.94 (0.9–0.98)	6.0E-03	9.0E-03	0.92 (0.88–0.98)	6.0E-03	1.0E-02	0.84 (0.73–0.97)	2.0E-02	3.0E-02

Each of the 59 prognostic regulators that were identified across the three datasets (Fig 3A) using univariate Cox PH modeling were selected for multivariate Cox PH adjustment incorporating clinicopathological data. Variables adjusted for were all available, non-redundant factors for each dataset as follows: METABRIC, patient age at diagnosis, disease stage, tumor grade, ER status (positive or negative), PR status (positive or negative), HER2 status (positive or negative), and non-surgical treatment status (whether the patient received treatment beyond surgery); Ur-Rehman and Vijver: patient age at diagnosis, tumor size, tumor grade, lymph node status (positive or negative), ER status (positive or negative), and non-surgical treatment status (whether the patient received treatment beyond surgery). P-values were adjusted for multiple comparisons into Q-values using the FDR method. 29 regulatory motifs met Q-value significance thresholds (< 0.01 for METABRIC, and < 0.05 for Ur-Rehman and Vijver, reflecting differences in statistical power) and are listed. Motifs are ordered by descending magnitude of the adjusted hazard ratio (HR) in the METABRIC dataset (second column). Survival information is disease-specific for METABRIC (n = 1,481), relapse-free for Ur-Rehman (n = 762), and overall for Vijver (n = 260), respectively.

## Discussion

Dysregulation of gene expression is integral to essentially all disease states, making the characterization of regulator activity of central importance in understanding pathophysiology. While past efforts have successfully introduced methods to infer regulator activity levels based on the expression levels of their target genes [31, 37], these methods necessarily require regulator target gene lists or binding profiles [8]. This makes the accuracy of target gene lists and binding profiles crucial for accurate regulatory activity inference and downstream analysis of its implications. As a regulator’s targets vary across contexts, regulator-characterizing experiments are expensive and noisy, and target gene list curation lacks stringency (if it is even pursued), achieving this accuracy across contexts is a challenge.

The icTAIR method introduced here addresses this challenge. By using contextual gene expression data to infer a regulator’s activity from the expression levels of an associated target gene list in that context, it can then analyze via spearman correlation the degree to which each gene’s expression level contributes to the inference of the regulator’s activity. The iterative removal of uncorrelated genes from the target gene list, re-inference of regulator activity, and re-correlation of each remaining target gene with the re-inferred activity progressively refines the target list until only genes that affect the regulator’s end activity score are included. As such, icTAIR distills target gene lists for contexts to greatly enhance their context-specific accuracy. Examining the consecutive output of the icTAIR algorithm shows this process at work (Fig 2): with repeated iterations, the regulator target gene lists stabilize (Fig 2A) and downstream analyses achieve parallel leaps in accuracy (Fig 2B and 2C).

Utilizing icTAIR’s output for downstream breast cancer survival analysis demonstrates the performance improvement and findings it makes achievable. The MSigDB c3 database of target gene lists, representing targets of the known evolutionarily conserved regulome in mammals [38], should itself be accurate and enable robust analysis. Nevertheless, icTAIR refinement of the database achieves an improvement in statistical significance of regulatory motif breast cancer survival HRs of roughly 7 orders of magnitude with a resultant enormous increase in identified prognostic regulatory motifs (Figs 3 vs. S1). Most impressive are the gains achieved for miRNAs (red dots, Figs 3B and S1). This is perhaps unsurprising, as miRNA targets tend to be predicted based on complementary sequence homology more than experimentally confirmed, suggesting that they may have the greatest opportunity for accuracy improvement. It is additionally noted that this analysis employed icTAIR refinement in a dataset distinct from those in which prognostic studies were undertaken, that these gains were seen in all datasets, and that the datasets were generated across seven independent studies using both one- and two- channel microarray platforms. The likelihood that the refined output and its results are thus artifacts of any given dataset, experimental system, or the icTAIR algorithm is improbable.

Further increasing confidence in the icTAIR-enabled analysis are specific result examples. For one, the uncovered prognostic effect of the ER regulatory motif matches the expected result, conferring a favorable prognosis in ER positive tumors and no effect on prognosis in ER negative ones (Fig 4). The ER regulatory motif iRASs are also remarkably lower in ER negative vs. ER positive samples. Continued examination of the results shows that E2F, HIF1, and MIR-19 associated regulatory motifs are uncovered as unfavorably prognostic in all datasets, results that reconfirm those of prior studies conducted by us and others using independent approaches and data sources [29, 34–36]. Beyond statistical and general icTAIR validation achieved with comparing Fig 3 to S2 Fig, these findings demonstrate the biological sensibility of the analysis’s output.

This confidence provides for continued exploration of the results and derivation of their meaning. Examining regulator prognosis by regulator type (Fig 3B) shows that miRNAs (red dots) overwhelmingly have positive HRs, indicating that general miRNA functional overexpression may be of broadly unfavorable breast cancer prognostic significance. Whether this is instigative (mechanistic of a worse outcome), reactionary (an induced, failing attempt by regulatory systems to reduce aberrant mRNA overexpression) or a mixture of the two requires further investigation. Turning to the relationships of the prognostic regulators, a regulatory network constructed of and among them (Fig 6) highlights their interconnectedness and their particularly central members (Fig 6A, sizes of spheres; Fig 6B, number of connecting lines). The FOXO4, E2F, TFAP4, TCF, and NFYA regulatory programs particularly stand out. In contrast, the miRNA programs tend to surround the periphery, although this may be artefactual and represent decreased information regarding their personal expression regulatory regions.

A detailed analysis of regulator prognostic significance when controlling for clinicopathological variables is perhaps of greatest clinical importance. Taking the 59 pan-dataset univariate prognostic regulatory motifs identified from univariate survival analysis (Fig 3A), multivariate adjustment for all available clinicopathological factors and a repeat of significance testing with multiple comparisons correction finds 29 regulatory motifs that remain prognostic in all datasets (Table 1). These motifs provide prognostic information for breast cancer beyond currently assessed factors and likely represent entirely new mechanistic pathways of tumorigenesis and potential avenues for therapy. Given past results, it is unsurprising and confirmatory that E2F-associated regulatory motifs are extensively repeated across the list. More intriguingly is the repetition of NFY regulatory motifs and their placement at the very top (largest magnitude of prognostic effect) of the cohort. This recasts the NFY target-to-regulator relationship to E2F and autoregulatory behavior (Fig 6B) in a new light. By others, the NFY TF family has been previously studied as a mediator of tissue invasion in granulocytes via regulation of cell-to-cell adhesion through induction of CD34 [39, 40], has known roles in angiogenesis [41], is negatively regulated by p53 [42], and has been implicated in colorectal adenocarcinoma formation and metastasis [43]. Still, to our knowledge the finding here is the first time NFY activity has been identified as dysregulated and of key prognostic importance in breast cancer.

In this study, we focused on MSigDB c3 regulator target gene list refinement and their implications for improving downstream analyses in breast cancer. A limitation of these gene lists is that target genes are predicted based on the presence of binding motifs in promoter proximal DNA regions for TFs and in the 3’UTR of mRNAs for miRNAs, without further experimental validation. Thus, these gene lists are generally associated with both high false positive (by lacking experimental validation) and false negative (by restricting all regulator activity to binding in target promoter regions) rates. However, we note that icTAIR is a flexible method that can take as input gene expression and target gene list data from any source to provide contextual refinement. There are numerous possibilities for this, not least of which is improving long-distance (enhancer) TF target gene prediction from ChIP-Seq data. We hope that icTAIR will help power many genomic-based analyses across many contexts going forward.

## Materials and Methods

### Dataset collection

Datasets used in this study were of two types: breast tumors gene expression and regulatory motif target gene lists. Tumor gene expression datasets were selected based on completeness of associated survival and clinicopathological data, sample size, and coverage of differing survival endpoints and microarray types. For the icTAIR contextual refinement dataset, TCGA level 3 breast cancer (BRCA) data covering 590 samples [44] were downloaded from the TCGA data portal (https://tcga-data.nci.nih.gov/tcga/). For survival analysis datasets, annotation and collation efforts from prior work [29, 45] led to the inclusion of the filtered, renormalized meta-analysis dataset from [32] (Ur-Rehman) containing 1,118 samples with survival and clinicopathological data from 5 independent datasets [46–50] and the dataset from [33] (Vijver) containing 260 samples with survival and clinicopathological data. Extending beyond prior efforts, the METABRIC dataset from [30] containing 1,992 samples was additionally selected due to its size and richness of clinicopathological data. Download sources were as follows: Ur-Rehman, the NCBI GEO database (www.ncbi.nlm.nih.gov/geo, GSE47561); Vijver, The Netherlands Cancer Institute (http://ccb.nki.nl/data/); METABRIC, the European Genome-phenome Archive (https://ega.crg.eu/studies/, study ID EGAS00000000083). Datasets were both one-channel (Ur-Rehman and METABRIC) and two-channel (TCGA and Vijver). Survival endpoints spanned relapse-free (Ur-Rehman), disease-specific survival (METABRIC), and overall survival (Vijver).

For the regulatory motif target gene lists, the MSigDB [26, 27] was queried and the c3 database of 825 TFs, miRNAs, and unclassified regulatory motif target gene lists selected as the most complete repository of evolutionarily-conserved [38] regulators available. Evolutionary conservation of regulators was desired to enrich for biological importance and accuracy of motifs. Target gene lists for each motif were downloaded from the GSEA repository (http://www.broadinstitute.org/gsea/msigdb/collections.jsp#C3). All data collection took place in December 2014, with dataset sizes reflecting all then-available samples / lists.

### The icTAIR algorithm

icTAIR requires as input two data sources, 1) a preliminary target gene list of a regulator and 2) a dataset of gene expression profiles of samples from a context of interest, and two parameters, I) the minimum allowable length of a target gene list and II) the minimum correlation threshold (0–1). An optional parameter, a maximum number of allowed iterations, may also be specified.

The first step of icTAIR is to input data sources 1) and 2) into the BASE algorithm for iRAS calculation (equivalent to the AC from [31], with the slightly-modified background function as introduced in [37]). Briefly, BASE works by sorting the gene expression data in descending order of expression level and generating two non-decreasing functions, the first to encapsulate the expression activity of the genes on the target gene list (foreground function) and the second to do the same for those that are not (background function). It then calculates the maximum division of the two functions to get a preliminary score. This preliminary score is similar in concept to the D-statistic of the Kolmogorov-Smirnov test and is representative of the expression activity of the target genes relative to background expression, with a higher preliminary score indicating relatively higher target gene activity and a lower preliminary score indicating relatively lower target gene activity. This preliminary score is then further normalized against the average of the absolute value of the preliminary scores from 1,000 randomly permuted target gene list to ultimately generate an iRAS for a given regulator. Of note, in this context icTAIR provides BASE the target gene list by creating a vector of all genes for the given genome (*e*.*g*., human RefSeq) and assigning a weight of 0 to the non-targets and 1 to the targets, *i*.*e*., the input is as a list ***g*** = [***g***_**1**_, ***g***_**2**_, ***g***_**3**_, …, ***g***_***j***_, …, ***g***_***n***_] where ***g***_***j***_ = 0 if a non-target, 1 if a target, and ***n*** = number of genes in the genome; this is so the target gene list matches the format of BASE’s binding affinity data (see [31]). Of further note, the expression level ***e*** of each gene ***g***_***j***_ for each sample ***s***_***i***_, ***e***_***j*,*i***_ is normalized to either its internal reference (for two-channel microarray expression data) or the median of its expression across the samples (for one-channel microarray expression data).

Once BASE generates the list of iRASs for the dataset of samples, icTAIR computes the spearman correlation coefficient *ρ* between each target gene *t*_*j*_’s expression and the sample iRASs, namely,

$$\rho \left(t_{j}\right)=1-\frac{6\sum_{i=1}^{s}d_{i}^{2}}{s\left(s^{2}-1\right)},$$

where *s* is the total number of samples and *d*_*i*_ is the difference in the rank parameter between the sorted values of *t*_*j*_*’s e*_*i*_ and the iRAS_*i*_. For each *t*_*j*_ in the target list, ***t*** = [***t***_**1**_, ***t***_**2**_, ***t***_**3**_, …, ***t***_***j***_, …, ***t***_***n***_], where *n* is the total number of target genes, icTAIR compares ***ρ***(***t***_***j***_) to the minimum correlation threshold parameter value. If the parameter is set as a *ρ* threshold (0–1), for all *t*_*j*_ for which ***ρ***(***t***_***j***_) is less than the threshold, its value in the gene list ***g*** is converted to 0 from 1 and it is dropped from the target list ***t***.

With these updated lists ***g*** and ***t***, iRASs are recalculated and the entire process repeated until either i) the length (*n*) of the target list ***t*** shrinks to the minimum allowable length parameter, ii) membership in ***t*** stabilizes, or (optional) iii) the maximum allowable iterations are reached.

The icTAIR algorithm has been implemented as a R function and can be downloaded from https://github.com/icTAIR.

### Univariate survival analyses

Using both the raw MSigDB c3 target gene lists and the output from icTAIR-refinement, BASE was employed to generate iRASs for each regulatory motif in the 3 overall survival analysis datasets (METABRIC, Ur-Rehman, and Vijver). Of note, while icTAIR does not require that its refinement be conducted in a gene expression dataset distinct from the datasets used for downstream analysis, this was done to reduce any potential dataset-specific source of confounding and because of a paucity of survival information for the TCGA data. For BASE, parameters were set at 1,000 permutations for all iRAS calculations and median normalization was implemented for the one-channel array datasets (METABRIC and Ur-Rehman).

Univariate Cox Proportional Hazards (PH) regression modeling was subsequently performed in each dataset for each regulatory motif’s iRAS. P-values of each hazard ratio (HR) for each motif were corrected for multiple comparisons (across all regulatory motifs) within each dataset into Q-values using the FDR method. Thresholds for statistical significance were set at Q-value < 1e-6 for METABRIC and Q-value < 1e-3 for Ur-Rehman and Vijver, reflecting differences in statistical power across these differently-sized and collected datasets and a desire to be highly conservative. Regulatory motifs that met these threshold criteria in all 3 datasets were deemed pan-dataset univariate prognostic. For visualization of selected pan-dataset univariate prognostic regulatory motifs, Kaplan-Meier (KM) survival curves were generated by dichotomizing the motifs’ associated iRASs around 0 into two groups for plotting. KM p-values were generated using the log-ranks test.

### Multivariate survival analyses

Regulatory motifs whose HRs met statistical significance thresholds in univariate Cox PH analyses in all three datasets (*i*.*e*., those deemed pan-dataset univariate prognostic) were chosen for follow-up clinicopathological multivariate Cox PH adjustment. Variables included for adjustment were all the non-redundant factors available for each dataset. For METABRIC, this included patient age at diagnosis, disease stage, tumor grade, ER status (positive or negative), PR status (positive or negative), HER2 status (positive or negative), and non-surgical treatment status (whether the patient received treatment beyond surgery); for Ur-Rehman and Vijver, this included patient age at diagnosis, tumor size, tumor grade, lymph node status (positive or negative), ER status (positive or negative), and non-surgical treatment status (whether the patient received treatment beyond surgery). As for univariate analyses, p-values of each motif’s multivariate-adjusted HR were corrected for multiple comparisons (across all adjusted motifs) within each dataset into Q-values using the FDR method. Thresholds for statistical significance were set at Q-value < 0.01 for METABRIC and Q-value < 0.05 for Ur-Rehman and Vijver, reflecting differences in statistical power across these datasets. Thresholds were lower than in univariate analyses due to a loss of power from multivariate correction and a decrease in sample count due to only including samples with full clinicopathological data. Sample sizes were 1,481 for METABRIC, 762 for Ur-Rehman, and 260 for Vijver.

For all survival analyses, survival endpoints were disease-specific survival in METABRIC, relapse-free survival in Ur-Rehman, and overall survival in Vijver. All work was performed using R software and its survival package, specifically the survreg(), survdiff(), and coxph() functions. Venn diagrams were constructed using the VennDiagram package.

### Prognostic regulator network analysis

icTAIR-refined univariate prognostic regulatory motif target gene lists were searched for the presence of other members in the MSigDB c3 regulome and used to assign regulator-to-target relationships: if a regulatory motif target gene list included another member of the MSigDB c3 regulome, it was deemed a regulator and the other member its target. This process was undertaken for all univariate prognostic regulatory motifs, searching among all other members in the MSigDB c3 regulome (*i*.*e*., 825 total) and within the restricted corpus of pan-dataset univariate prognostic regulatory motifs only. Regulator-target relational maps were subsequently constructed by referring to the annotated TFs of motifs. Due to the limitations of the unclassified regulatory motif data, these motifs were necessarily excluded from the more restricted analysis. All motifs redundant on a regulator were collapsed down to the regulator level.

## Supporting Information

S1 Fig Cox PH results in the Ur-Rehman and Vijver datasets based on 59 significant regulators. (A) Volcano plot for Ur-Rehman dataset with each dot a unique regulator color-coded by type and plotted based on its HR and Q-value. Green, TF; Red, miRNA; Cyan, unclassified regulator motif. Given the dot distribution, it is seen that the TFs tend to have more favorable prognostic implications that do miRNAs. (B) Repeat analyses for the Vijver datasets show similar results.
(TIF)

S2 Fig Survival analysis using MSigDB c3 target gene lists without iCTIR refinement. All results are inferior as compared to Fig 3. Of particular note is the lack of any prognostic discernment in the Vijver dataset (no Vijver results) and the much-reduced statistical significance of all results. No pan-dataset prognostic regulators are identified. (A) Venn diagrammatic breakdown of significant regulators across the datasets. All regulators that passed Q-value significance thresholds (1e-06 for METABRIC and 1e-03 for Vijver and Ur-Rehman, reflecting differences in statistical power) are included. (B) Volcano plot of the regulators’ Cox PH results, METABRIC Dataset. The x-axis indicates the Hazard Ratio (HR) and the y-axis the FDR–corrected degree of significance (Q-value), scaled by a -log10 transformation. Each dot is a regulator colored by type: green, TF; red, miRNA; cyan, unclassified regulator motif. The horizontal line indicates a Q-value cutoff of 1x10^−6^. (C) Pair-wise analysis of survival effect size concordance between datasets. Left: Ur-Rehman vs. METABRIC; middle: Vijver vs. METABRIC; right: Vijver vs. Ur-Rehman. Each dot represents a HR coordinate (HR in first dataset, HR in second dataset) for each statistically significant regulator shared between the indicated datasets. Dot coloring is by type: green, TF; red, miRNA; cyan, unclassified regulator motif.
(TIF)

S1 Table The 59 pan-dataset univariate prognostic regulatory motifs. (XLSX)

S2 Table The 59 pan-dataset univariate prognostic regulatory motifs with clinicopathological multivariate adjustment (Ur-Rehman dataset). (XLSX)

S3 Table The 59 pan-dataset univariate prognostic regulatory motifs with clinicopathological multivariate adjustment (METABRIC dataset). (XLSX)

S4 Table The 59 pan-dataset univariate prognostic regulatory motifs with clinicopathological multivariate adjustment (Vijver dataset). (XLSX)
