# Supplementary figures and images for: Contextual Refinement of Regulatory Targets Reveals Effects on Breast Cancer Prognosis of the Regulome
Source: PLoS Comput Biol. 2017 Jan 19;13(1):e1005340. doi: 10.1371/journal.pcbi.1005340 (PMC5289608; doi:10.1371/journal.pcbi.1005340)

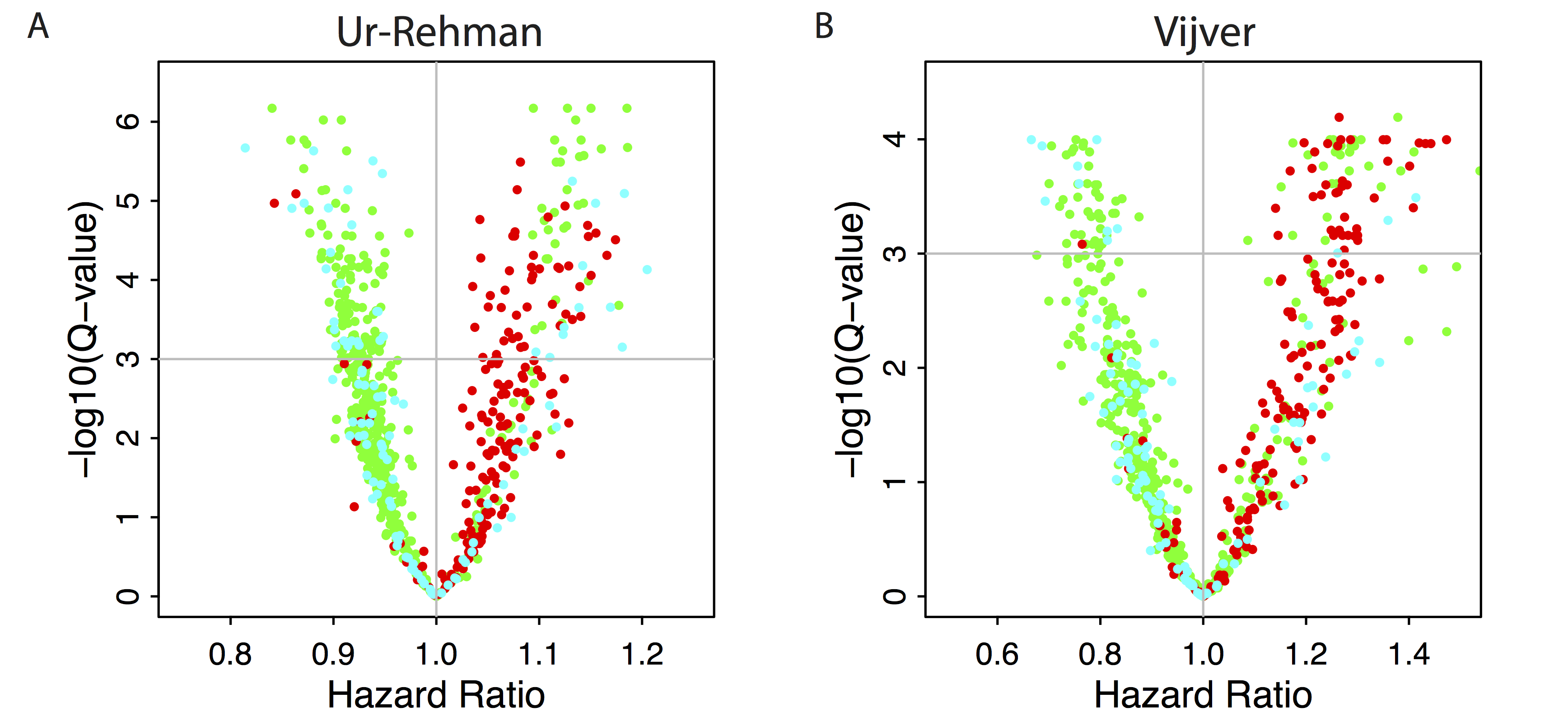

Supplement: S1 Fig — (A) Volcano plot for Ur-Rehman dataset with each dot a unique regulator color-coded by type and plotted based on its HR and Q-value. Green, TF; Red, miRNA; Cyan, unclassified regulator motif. Given the dot distribution, it is seen that the TFs tend to have more favorable prognostic implications that do miRNAs. (B) Repeat analyses for the Vijver datasets show similar results. (TIF) [file pcbi.1005340.s001.tif]

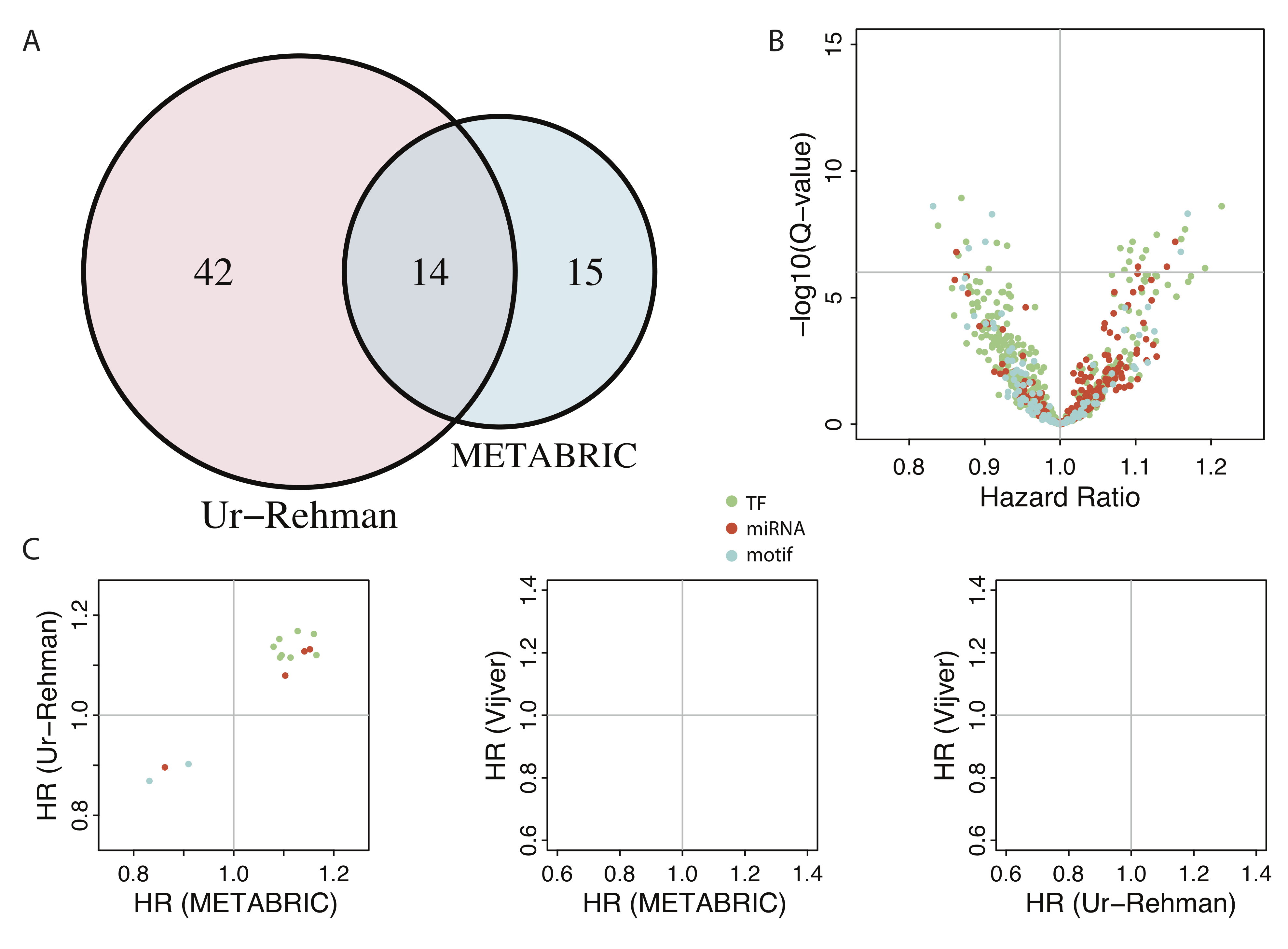

Supplement: S2 Fig — All results are inferior as compared to Fig 3. Of particular note is the lack of any prognostic discernment in the Vijver dataset (no Vijver results) and the much-reduced statistical significance of all results. No pan-dataset prognostic regulators are identified. (A) Venn diagrammatic breakdown of significant regulators across the datasets. All regulators that passed Q-value significance thresholds (1e-06 for METABRIC and 1e-03 for Vijver and Ur-Rehman, reflecting differences in statistical power) are included. (B) Volcano plot of the regulators’ Cox PH results, METABRIC Dataset. The x-axis indicates the Hazard Ratio (HR) and the y-axis the FDR–corrected degree of significance (Q-value), scaled by a -log10 transformation. Each dot is a regulator colored by type: green, TF; red, miRNA; cyan, unclassified regulator motif. The horizontal line indicates a Q-value cutoff of 1x10^−6^. (C) Pair-wise analysis of survival effect size concordance between datasets. Left: Ur-Rehman vs. METABRIC; middle: Vijver vs. METABRIC; right: Vijver vs. Ur-Rehman. Each dot represents a HR coordinate (HR in first dataset, HR in second dataset) for each statistically significant regulator shared between the indicated datasets. Dot coloring is by type: green, TF; red, miRNA; cyan, unclassified regulator motif. (TIF) [file pcbi.1005340.s002.tif]
